# Introducing a new pleosporalean family Sublophiostomataceae fam. nov. to accommodate *Sublophiostoma* gen. nov.

**DOI:** 10.1038/s41598-021-88772-w

**Published:** 2021-05-04

**Authors:** Sinang Hongsanan, Rungtiwa Phookamsak, Ishani D. Goonasekara, Kasun M. Thambugala, Kevin D. Hyde, Jayarama D. Bhat, Nakarin Suwannarach, Ratchadawan Cheewangkoon

**Affiliations:** 1 Department of Entomology and Plant Pathology, Faculty of Agriculture, Chiang Mai University, Chiang Mai, 50200 Thailand; 2 CAS Key Laboratory for Plant Biodiversity and Biogeography of East Asia (KLPB), Kunming Institute of Botany, Chinese Academy of Science, Kunming, 650201 Yunnan China; 3 Center of Excellence in Fungal Research, Mae Fah Luang University, Chiang Rai, 57100 Thailand; 4 School of Science, Mae Fah Luang University, Chiang Rai, 57100 Thailand; 5 No. 128/1-J, Azad Housing Society, Curca, P.O., Goa Velha, 403108 India; 6 CIFOR-ICRAF China Program, World Agroforestry (ICRAF), Kunming, 650201 Yunnan China; 7 Genetics and Molecular Biology Unit, Faculty of Applied Sciences, University of Sri Jayewardenepura, Gangodawila, Nugegoda Sri Lanka; 8 Department of Plant and Molecular Biology, Faculty of Science, University of Kelaniya, Kelaniya, Sri Lanka; 9 Honghe Center for Mountain Futures, Kunming Institute of Botany, Chinese Academy of Sciences, Honghe County, 654400 Yunnan China; 10 Centre for Mountain Futures (CMF), Kunming Institute of Botany, Kunming, 650201 Yunnan China; 11 Research Center of Microbial Diversity and Sustainable Utilization, Chiang Mai University, Chiang Mai, 50200 Thailand; 12 Department of Biology, Faculty of Science, Chiang Mai University, Chiang Mai, 50200 Thailand

**Keywords:** Microbiology, Fungi, Fungal physiology

## Abstract

Collections of microfungi on bamboo and grasses in Thailand revealed an interesting species morphologically resembling *Lophiostoma*, but which can be distinguished from the latter based on multi-locus phylogeny. In this paper, a new genus, *Sublophiostoma* is introduced to accommodate the taxon, *S. thailandica* sp. nov*.* Phylogenetic analyses using combined ITS, LSU, RPB2, SSU, and TEF sequences demonstrate that six strains of the new species form a distinct clade within Pleosporales, but cannot be assigned to any existing family. Therefore, a new family Sublophiostomataceae (Pleosporales) is introduced to accommodate the new genus. The sexual morph of Sublophiostomataceae is characterized by subglobose to hemisphaerical, ostiolate ascomata, with crest-like openings, a peridium with cells of *textura angularis* to *textura epidermoidea*, cylindric-clavate asci with a bulbous or foot-like narrow pedicel and a well-developed ocular chamber, and hyaline, fusiform, 1-septate ascospores surrounded by a large mucilaginous sheath. The asexual morph (coelomycetous) of the species are observed on culture media.

## Introduction

Pleosporales Luttr. ex M.E. Barr is the largest order of Dothideomycetes O.E. Erikss. & Winka^1–8^. This order was invalidly introduced by Luttrell^9^ and subsequently validated by Barr^10^, based on the family Pleosporaceae Nitschke and its type species *Pleospora herbarum* (Pers.) Rabenh^11^. Lumbsch & Huhndorf^12^ listed 28 families and 175 genera in Pleosporales, while 12 genera were listed as Pleosporales, genera *incertae sedis*. Classification of these families was mainly based on morphological characters, along with support from molecular data^2,4,13,14^. Zhang et al.^8^ accepted 28 families in Pleosporales based on morphology and phylogeny, while Hyde et al.^2^ listed 41 families in this order. Wijayawardene et al.^5^ listed 39 families under Pleosporales, and 49 as Pleosporales genera *incertae sedis*. Hongsanan et al.^1^ accepted 91 families in Pleosporales. The order consists of two suborders, Pleosporineae and Massarineae. Zhang et al.^8^ reported that Pleosporineae comprised 28 families with 105 genera, but there were taxonomic agreements between morphology and the new phylogenetic arrangements. The subclass Pleosporineae is characterized by broadly to narrowly oblong ascomata, downwardly growing pseudoparaphyses, and bitunicate asci with uni- to multi-septate ascospores^2^. The suborder Massarineae is characterized by immersed or superficial ascomata, cylindrical asci with a short pedicel and uni- to multi-septate ascospores^2^ and comprises 12 families^15^.

Species of Pleosporales are highly diverse, occurring as saprobes on dead plant materials in both aquatic and terrestrial habitats, while a large proportion, especially the asexual species, inhabit living plants as pathogens^2,8,16–19^. Therefore, it is necessary to collect additional taxa from diverse localities, isolate them in culture and analyse their DNA to resolve taxonomic placements. In this paper, we introduce a novel family Sublophiostomataceae in Pleosporales, to accommodate the newly introduced genus *Sublophiostoma,* based on its type *Sublophiostoma thailandica*, complete with illustrations and descriptions. The new taxa are listed in the webpage dothideomycetes.org^20^.

## Results

### Molecular analyses

**Figure 1:**
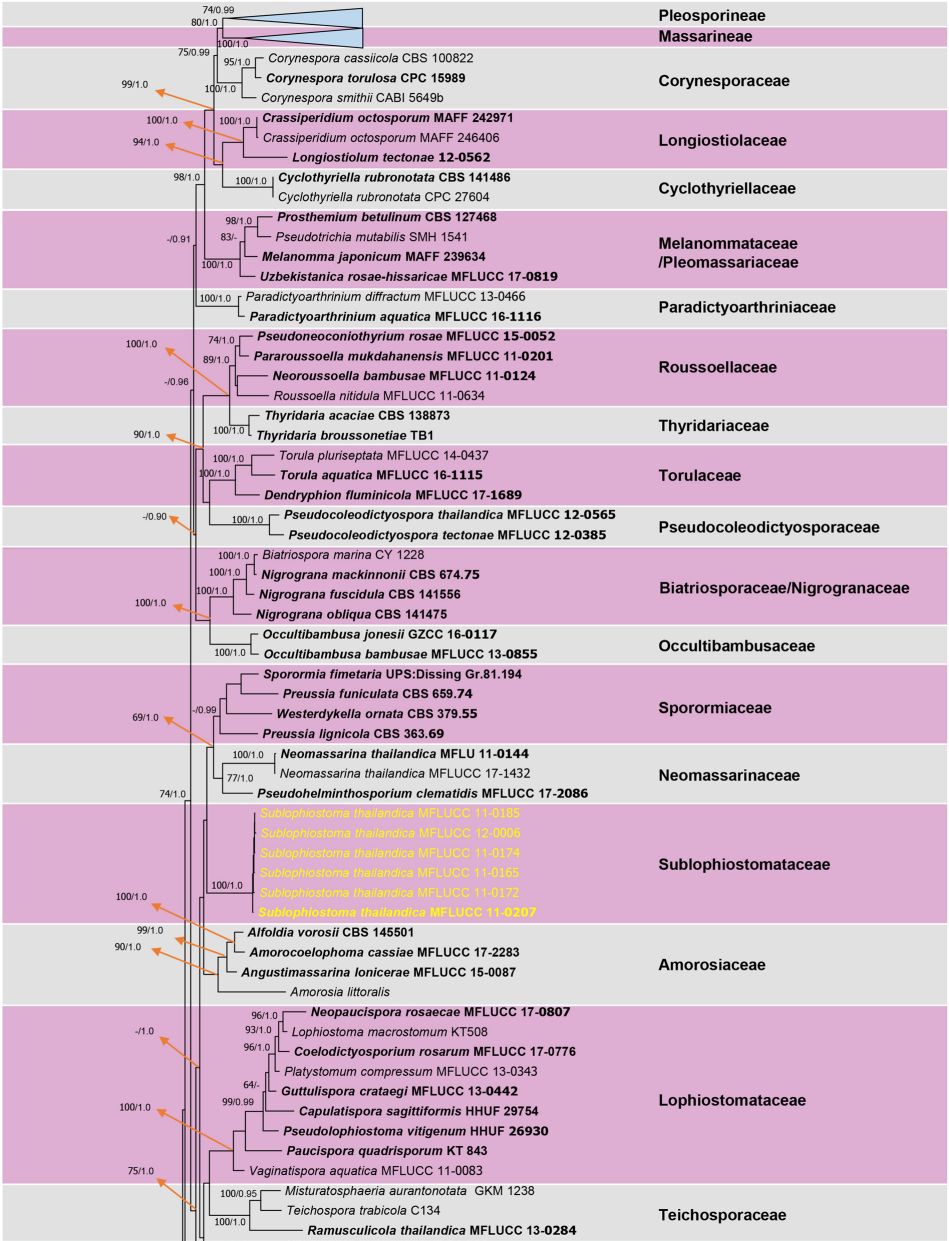

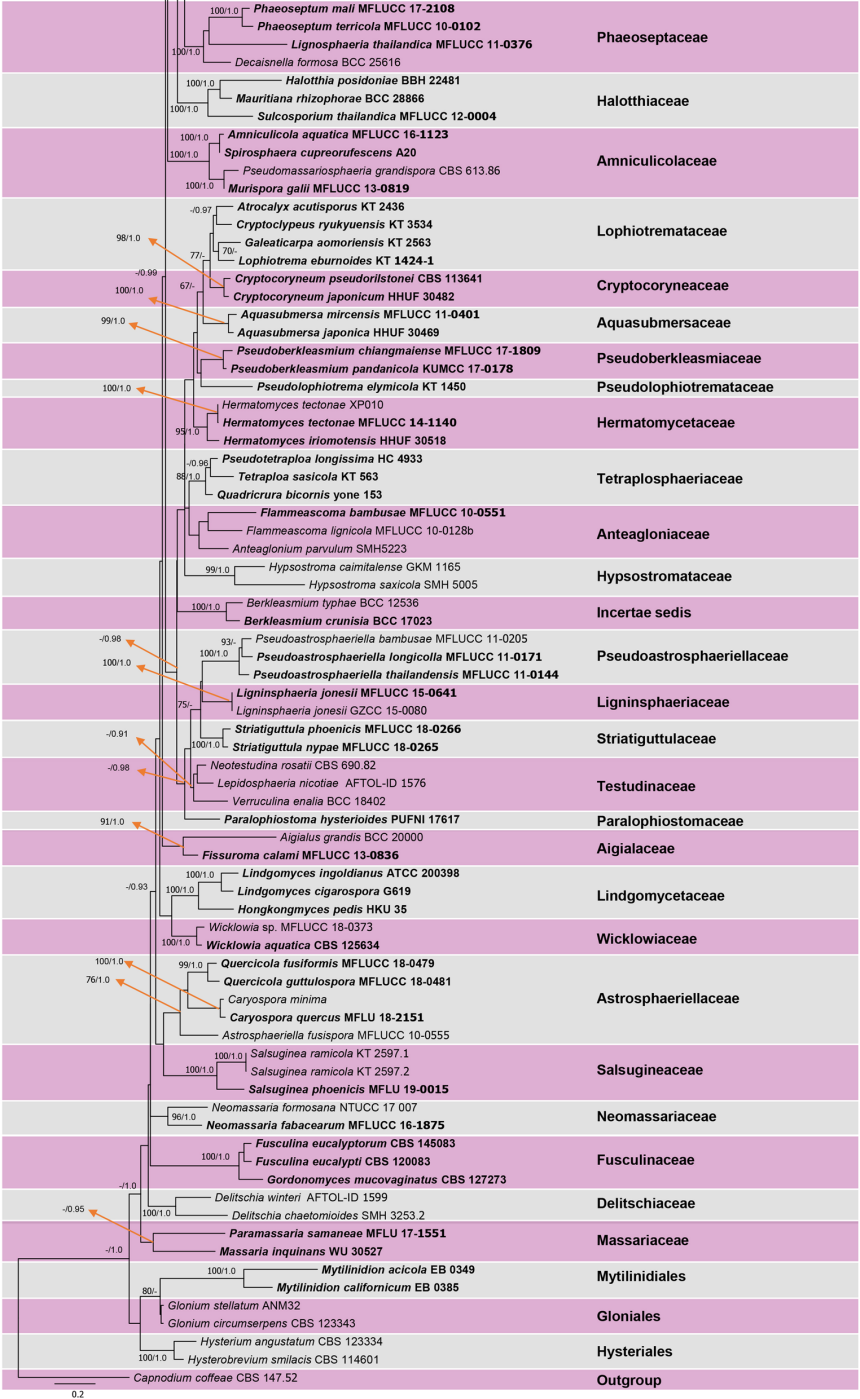
Resulting phylogram of maximum-likelihood analysis based on a combined ITS, LSU, RPB2, SSU and TEF sequence data. Maximum likelihood bootstrap support values (ML) equal or higher than 60% and Bayesian probability values (BYPP) equal to or above 0.90 are given at the nodes (ML/ BYPP). The tree is rooted to *Capnodium coffeae* (CBS 147.52). Newly introduced strains are indicated in yellow and ex-type strains in bold.

A combined dataset of ITS, LSU, RPB2, SSU, and TEF sequence data was used to evaluate the phylogenetic placement of the new taxa. The ML and BI analyses resulted in a almost similar topology with no significant difference. Therefore the phylogram resulting from the ML analysis was selected to show the phylogenetic placement of the newly introduced family and its related families in the order Pleosporales (Fig. 1). The alignment of combined genes analyses comprised 235 taxa with representative strains of the all families in Pleosporales. The new strains grouped together (100% ML and 1.00 BYPP support, Fig. 1) as a distinct clade adjacent to Neomassarinaceae and Sporormiaceae, with low support from both analyses (Fig. 1).

**Figure 2:**
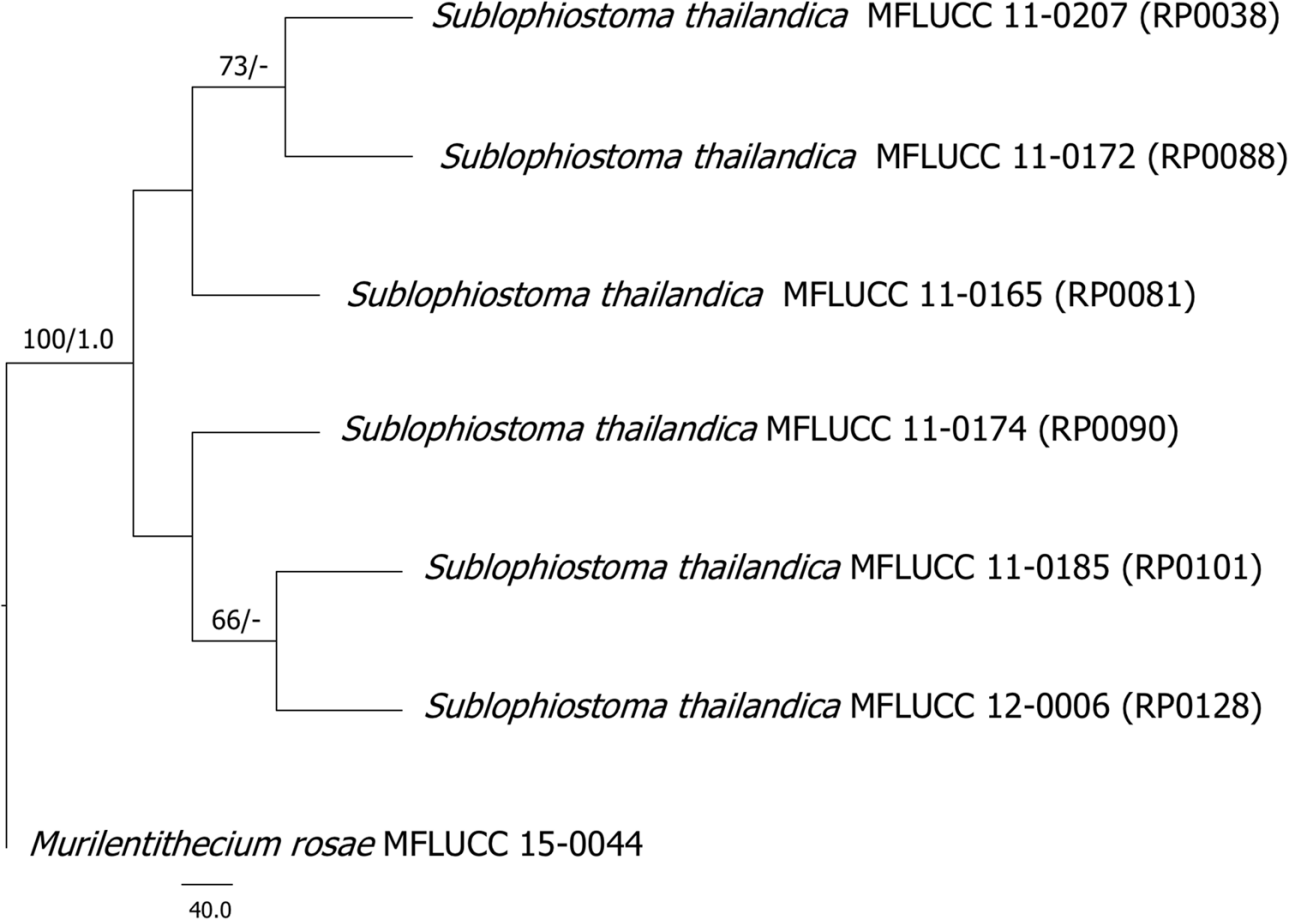
The first of 1,000 trees from maximum parsimony analysis of combined ITS, LSU, SSU, RPB2 and TEF sequence data for the species of *Sublophiostoma*. Maximum parsimony bootstrap support values (MP) equal or higher than 60% and Bayesian probability values (BYPP) equal to or above 0.90 are given at the nodes (MP/ BYPP). The tree is rooted to *Murilentithecium rosae* (MFLUCC 15–0044).

We introduce six strains of *Sublophiostoma* as a single species and note that they have intraspecific variation of RPB2. There is no report about intraspecific variation in the families Neomassarinaceae and Sporormiaceae which are related to Sublophiostomataceae in our phylogenetic tree. A comparison of ITS of the six new isolates showed that they have a single base pair difference and have less than ten base pair differences in TEF. However, they have more than ten base pair differences in RPB2. Therefore, an MP analysis which included only strains of *Sublophiostoma* and *Murilentithecium rosae* (MFLUCC 15–0044) as outgroup taxon was conducted for a combined dataset of the ITS, LSU, SSU, TEF and RPB2 (Fig. 2).

#### Sublophiostomataceae Hongsanan, Phookamsak, K.D. Hyde & Cheewangkoon, fam. nov

Index Fungorum number: IF557974; Facesoffungi number: FoF09401.

Generic type: *Sublophiostoma* Phookamsak, Hongsanan & K.D. Hyde.

Notes: The family Sublophiostomataceae is introduced to accommodate the monotypic genus *Sublophiostoma*. The sexual morph of this family closely resembles Lophiostomataceae (*Lophiostoma*, *Pseudolophiostoma* and *Vaginatispora*) in having immersed to erumpent, subglobose, ostiolate ascomata with a crest-like or slit-like apex, cylindrical to clavate, pedicellate asci with an ocular chamber and fusiform ascospores with narrow, acute ends. However, members of Lophiostomataceae have uni- to multi-septate or muriform ascospores without a sheath or sheath drawn out at the ends, while Sublophiostomataceae has 1-septate ascospores surrounded by a large mucilaginous sheath. The reported asexual morph of Lophiostomataceae is coelomycetous, producing cylindrical to ellipsoid, aseptate to multi-septate or distoseptate, hyaline, light brown to brown conidia^2,21–23^. The asexual morph of Sublophiostomataceae is coelomycetous, producing subglobose to oblong, hyaline, aseptate, smooth-walled conidia. Sublophiostomataceae also resembles Amniculicolaceae (*Amniculicola*), Amorosiaceae (*Angustimassarina*), Bambusicolaceae (*Bambusicola*), Teichosporaceae (*Ramusculicola*), Gloniaceae (*Glonium*) and Tetraplosphaeriaceae (*Tetraploa*) in having similar asci and ascospore characteristics^2,23^. There are many genera/species with quite similar asexual morph such as *Ramusculicola thailandica* Thambug. & K.D. Hyde and *Trematosphaeria pertusa* Fuckel. However, Sublophiostomataceae is phylogenetically distinct from the above mentioned species (Fig. 1).

#### Description of *Sublophiostoma* Phookamsak, Hongsanan & K.D. Hyde, gen. nov

Index Fungorum number: IF557972; Facesoffungi number: FoF09402.

Etymology: The generic epithet “*Sublophiostoma*” refers to the resemblance of *Lophiostoma*.

*Saprobic* on grasses and bamboo (Poaceae). **Sexual morph**: *Ascomata* solitary to gregarious, scattered, immersed to semi-immersed or erumpent, visible as dark ellipsoidal spots on the host surface, uni-loculate, coriaceous, black, subglobose, hemisphaerical to lenticular, glabrous, centrally ostiolate with minute papilla beneath the host tissue, with crest-like opening. *Peridium* thick at upper lateral part and thin at the base, composed of several layers of cells, with outer layers of thick, dark brown layers of cells of *textura angularis* and inner layers comprising hyaline, somewhat flattened cells of *textura angularis* to *textura epidemoidea*. *Hamathecium* comprising dense, septate, branched trabeculate pseudoparaphyses, embedded in a gelatinous matrix. *Asci* 8-spored, bitunicate, fissitunicate, cylindric-clavate, rounded at the apex, with a small bulbous pedicel, with well-developed ocular chamber. *Ascospores* uni to bi-seriate, partially overlapping, hyaline, fusiform, 1-septate, constricted at the septum, with each cell swollen near the septum, becoming acute at the ends, guttulate, smooth-walled, surrounded by a large mucilaginous sheath. **Asexual morph**: Coelomycetous, produced on bamboo pieces on WA after 8 weeks, appearing as black, punctiform, globose structures, covered by grey to dark grey vegetative mycelium. *Conidiomata* solitary or aggregated, pycnidial, obpyriform. *Conidiomata walls* comprising of outer, brown cells of *textura globulosa* to *textura angularis* and inner, hyaline cells of *textura angularis*. *Conidiophores* reduced to conidiogenous cells. *Conidiogenous cells* hyaline, phialidic, integrated. *Conidia* aseptate, smooth-walled, subglobose to oblong, with two large guttules.

Type species: ***Sublophiostoma thailandica*** Phookamsak, Hongsanan, Goonas. & K.D. Hyde.

Notes: Morphologically, *Sublophiostoma* resembles the genera in Lophiostomataceae such as *Lophiostoma*, *Pseudolophiostoma* and *Vaginatispora* based on its ascomata and asci. However, *Sublophiostoma* differs from these genera in having peridium with *textura angularis* to *textura epidermoidea*, 1-septate ascospores surrounded by wide sheath, while *Lophiostoma*, *Pseudolophiostoma* and *Vaginatispora* have peridium with *textura angularis* cells, 1- to multi-septate ascospores, lacking a mucilaginous sheath or surrounded by a thin sheath which is drawn out at the ends^23^. Phylogenetic analyses (Fig. 1) demonstrate that *Sublophiostoma* does not cluster with Lophiostomataceae and forms a distinct clade which cannot be assigned to any known family in Pleosporales. Therefore, the new family, Sublophiostomataceae is established to accommodate this genus.

#### Description of *Sublophiostoma thailandica* Phookamsak, Hongsanan, Goonas. & K.D. Hyde, sp. nov

**Figure 3:**
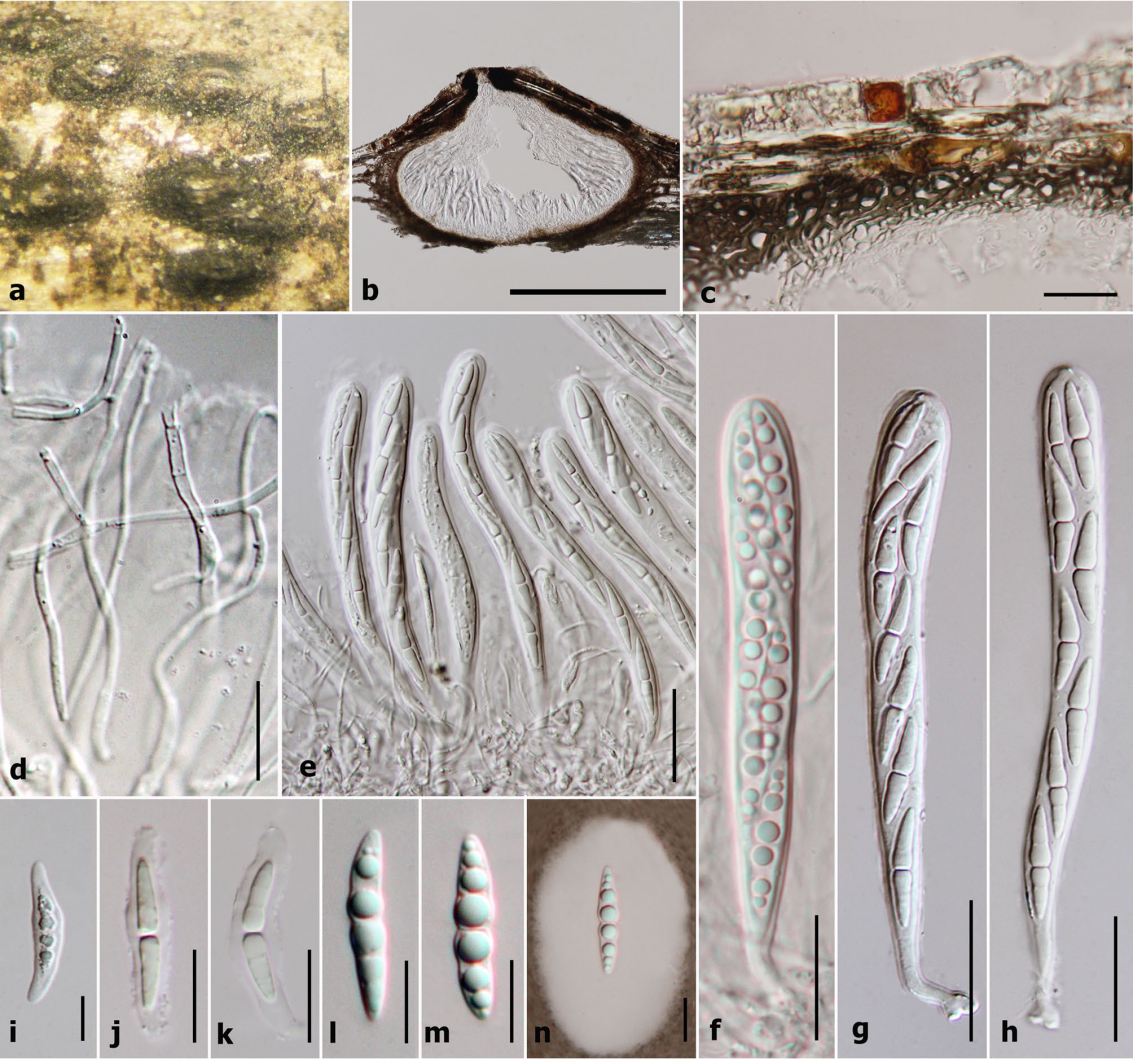
Sexual morph characters of *Sublophiostoma thailandica* MFLU 11–0158. (**a**) Appearance of ascomata on host. (**b**) Section of ascoma. (**c**) Peridium. (**d**) Trabeculate pseudoparaphyses. (**e–h**) Asci. (**i–m**) Ascospores. (**n**) Appearance of mucilaginous sheath surrounding young ascospore (stained in Indian ink). Scale Bars: **b** = 100 µm, **c**, **d** = 20 µm, **e–i**, **l–n** = 10 µm, **j**, **k** = 5 µm.

**Figure 4:**
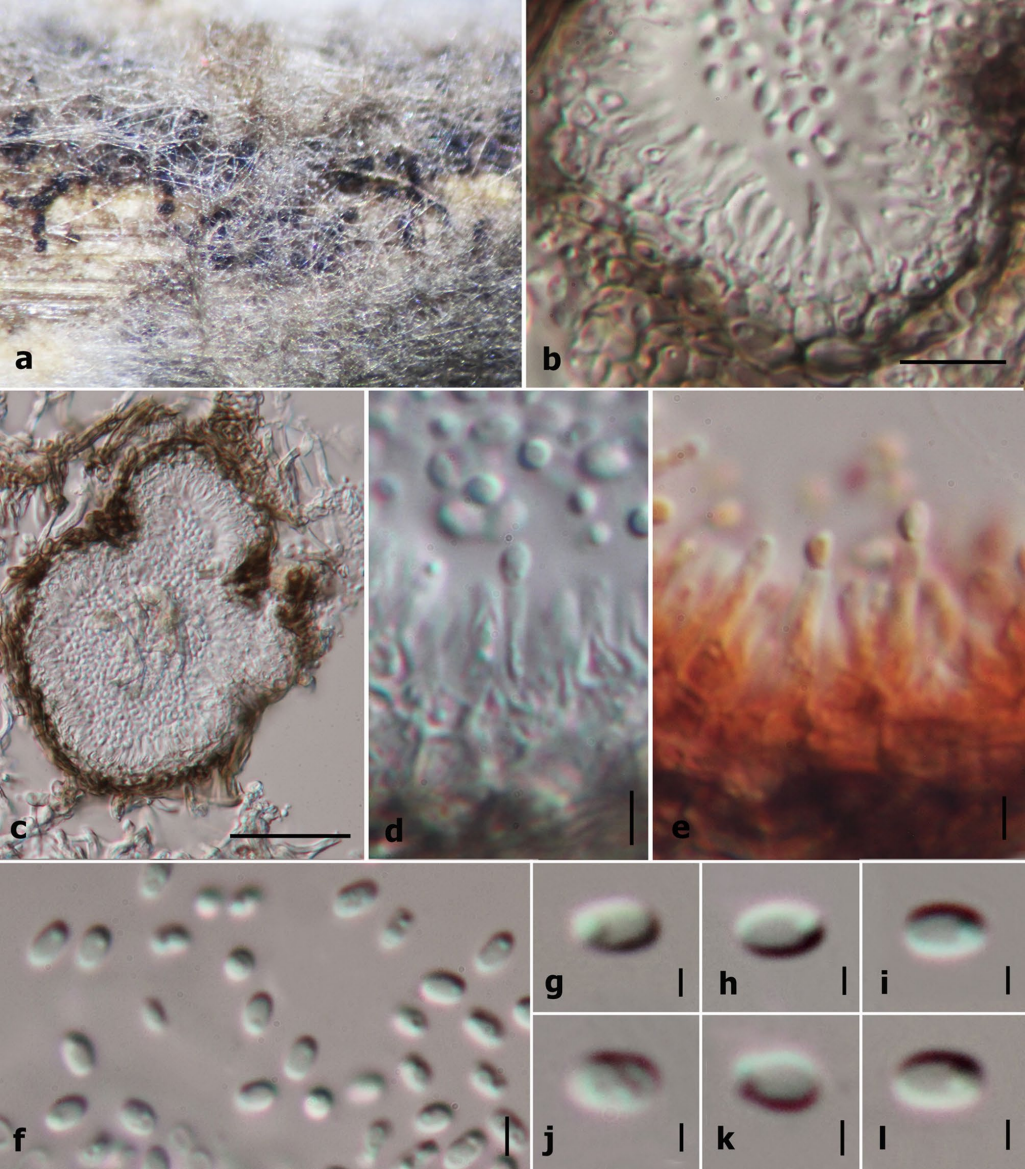
Asexual morph characters of *Sublophiostoma thailandica* MFLU 11–0158. (**a**) Conidiomata on bamboo pieces on WA after 8 weeks. (**b**) Conidioma wall. (**c**) Section of conidioma. (**d)** Conidiogenous cells. **(e**) Conidiogenous cells stained in congo red. (**f–l**) Conidia. Scale bars: **b** = 10 µm, **c** = 50 µm, **d, e, f** = 2 µm, **g–l** = 1 µm.

Index Fungorum number: IF557973; Facesoffungi number: FoF09403 (Figs. 3, 4).

Etymology: The specific epithet “*thailandica*” refers to the country from which the species was collected.

Holotype: MFLU 11–0158.

*Saprobic* on grasses and bamboo (Poaceae). **Sexual morph:** *Ascomata* 450–600 µm high × 135–185 µm diam. (*x̅* = 505 × 160 µm, n = 10), solitary to gregarious, scattered, immersed to semi-immersed or erumpent, visible as dark ellipsoidal spots on host surface, uni-loculate, coriaceous, black, subglobose, hemisphaerical to lenticular, glabrous, central ostiole with minute papilla beneath the host tissue, crest-like opening. *Peridium* 13–30 µm wide, thick at upper lateral part and thinner at the base, composed of several layers of cells, with outer layers of thick, dark brown layers of cells of *textura angularis* and inner layers comprising hyaline, somewhat flattened cells of *textura angularis* to *textura epidemoidea*. *Hamathecium* comprising 0.6–1.5 µm wide, dense, septate, branched trabeculate pseudoparaphyses, embedded in a gelatinous matrix. *Asci* 85–105 µm × 8.5–10.5 µm (*x̅* = 95 × 9 µm, n = 30), 8-spored, bitunicate, fissitunicate, cylindric-clavate, rounded at apex, with a small bulbous pedicel, with well-developed ocular chamber. *Ascospores* 20.4–25.5 × 3.5–5.6 µm (*x̅* = 23.7 × 4.5 µm, n = 30), uni to bi-seriate, partially overlapping, hyaline, fusiform, 1-septate, constricted at the septum, each cell wider near the septum, becoming acute at the ends, guttulate, smooth-walled, surrounded by a large mucilaginous sheath. **Asexual morph:** Coelomycetous, produced on bamboo pieces on WA after 8 weeks, appearing as black, punctiform, globose structures, covered by grey to dark grey vegetative mycelium. *Conidiomata* solitary or aggregated, pycnidial, obpyriform. *Conidiomata walls* 5–10 µm (*x̅* = 7 µm, n = 14) in thickness, comprising outer, brown cells of *textura globulosa* to *textura angularis* and inner, hyaline cells of *textura angularis*. *Conidiophores* reduced to conidiogenous cells. *Conidiogenous cells* 5.7–7.7 µm (*x̅* = 6.7 µm, n = 13) in length, phialidic, with narrow periclinal thickening, hyaline, integrated. *Conidia* 1.8–2.6 × 1.1–1.8 µm (*x̅* = 2.4 × 1.4 µm, n = 20), hyaline, aseptate, smooth-walled, subglobose to oblong, with large guttules.

*Colonies* on PDA medium showing slow growth, 30–35 mm diam. after 4 weeks at 25–30 °C, white to pale yellow at the magins, white to grey in the center, with dark grey turfing in the middle of the colony; reverse white at the outer margin, more grey towards the inner area, becoming yellowish-grey in the center, dense, irregular, slightly raised to umbonate, dull with undulate edge, velvety, not produced pigmentation on agar.

*Material examined:* THAILAND, Chiang Mai Prov., Chom Tong District, Doi Inthanon, on dead stems of grass, 16 November 2010, R. Phookamsak RP0038 (MFLU 11–0158, **holotype**), ex-type living culture, MFLUCC 11–0207, *ibid*. on dead branches of bamboo, 6 October 2010, R. Phookamsak, RP0081 (MFLU 11–0201), living culture MFLUCC 11–0165; on dead stem of *Thysanolaena maxima* Kuntze, 6 October 2010, R. Phookamsak, RP0088 (MFLU 11–0208), living culture, MFLUCC 11–0172; on dead stem of *Thysanolaena maxima*, 16 November 2010, R. Phookamsak. RP0090 (MFLU 11–0210), living culture MFLUCC 11–0174; on dead stem of *Thysanolaena maxima*, 16 October 2010, R. Phookamsak, RP0101 (MFLU 11–0221), living culture, MFLUCC 11–0185; Chiang Rai Prov., Muang District, Khun Korn Waterfall, on dead stem of *Thysanolaena maxima*, 21 June 2011, R. Phookamsak, RP0128 (MFLU 11–0245), living culture MFLUCC 12–0006.

## Discussion

Phylogenetic analyses clearly indicate that six strains of *Sublophiostoma thailandica* form a distinct clade outside the suborders Massainae and Pleosporinae, but within Pleosporales (Fig. 1). The clade of *Sublophiostoma* is phylogenetically closely related to Neomassarinaceae and Sporormiaceae (Fig. 1). However, *Sublophiostoma* differs from Neomassarinaceae in having black, subglobose, hemisphaerical to lenticular, glabrous ascomata, a peridium of cells of *textura angularis* to *textura epidemoidea* at the inner layers and cylindric-clavate asci with a long pedicel. Genera in Neomassarinaceae have light brown to brown, coriaceous ascomata, a peridium with cells of *textura angularis*, cylindrical to cylindric-clavate asci, with a short pedicel, and a narrow sheath or surrounded by hyaline gelatinous sheath^1,24^. Sporormiaceae differs from *Sublophiostoma* in having globose to pyriform, perithecioid or cleistothecioid, ascolocular pseudothecia, membraneous or coriaceous ascomata, with or without an ostiolar canal, a peridium of darkly pigmented cells of *textura angularis*, clavate, globose or cylindrical asci and dark brown, usually septate and poly-celled, muriform ascospores, with oblong, suboboviod, hyaline to brown, 1-septate conidia in its asexual morph^1^. *Sublophiostoma* shares similar characteristics with Lophiostomataceae, but is different in having *textura angularis* to *textura epidermoidea* cells in the peridium, and ascospores surrounded by a thick mucilaginous sheath. Most species of Lophiostomataceae have been reported from dicotyledons and are saprobic on twigs and bark. However, taxa of Sublophiostomataceae herein is discovered from stems or branches of monocotyledons (bamboo and grass). Therefore, we introduce a new family, Sublophiostomataceae in Pleosporales based on morphology and phylogeny (Figs. 1, 2, 3, 4) to accommodate the monotypic genus *Sublophiostoma*.

Hongsanan et al.^1^ stated that the nature of the pseudoparaphyses (cellular or trabeculate, sensu Liew et al.^25^) should be considered when discussing new families in Dothideomycetes. In Sublophiostomataceae the pseudoparaphyses are clearly trabeculate (Fig. 1), in Neomassarinaceae they are cylindrical to filiform, septate, branching, cellular pseudoparaphyses and in Lophiostomataceae they are septate, long, hyaline, anastomosing and branched, cellular pseudoparaphyses, embedded in gelatinous matrix between and above the asci.

All the strains of *Sublophiostoma thailandica* were found in Chiang Mai and Chiang Rai Provinces which are located in the north of Thailand and therefore we cannot conclude whether members of Sublophiostomataceae are distributed in other parts of Thailand or worldwide. More taxa of this genus from diverse localities should be studied to understand the distribution of Sublophiostomataceae.

Considering the MP tree in Fig. 2 and number of base pair differences, it can be postulated that the six strains of *Sublophiostoma thailandica* could represent a species complex. Within the ITS regions, MFLUCC 11–0174 strain and MFLUCC 11–0185 strain have one nucleotide difference (< 1%) from MFLUCC 11–0207, MFLUCC 11–0165 and MFLUCC 11–0172. Across the TEF region, there are less than ten base pair differences (< 1%). Thus, for the time being, we considered the six strains as belonging to the same species, *Sublophiostoma thailandica* following the guidelines in Jeewon & Hyde^26^. Even the phylogenetic tree (Fig. 1) does support establishment of one species as all strains cluster together. However, it is worth mentioning that these six strains have more than ten base pair differences in RPB2. The latter gene region is well known for its DNA sequence variability and one can speculate that the above six strains could represent more than one species. Our MP tree (Fig. 2) depicts that these six strains can be segregated into 2 subclades but there is no support. These six strains are also slightly different in ascus shape and size and the sheath surrounding the ascospores but these could also be minor phenotypic variation within one species, a common phenomenon which happens across many fungi which are subjected to different in vitro environmental conditions. In order to consider them as distinct species or genetic variants of the same species, further in-depth analyses are needed.

The order Pleosporales presently comprises two suborders which are Massarineae and Pleosporinae, as well as many unresolved clades^1,2,8,13,19,23^. Many of these unresolved or unclear lineages have low bootstrap support, which is probably due to limited taxon-sampling^9,27^. An assessment of the morphological characters which is the traditional way of classifying fungi and DNA based phylogenetic analyses are important in determining the status of taxa and could help to resolve some of the taxonomic confusions that presently exist in Pleosporales.

## Materials and methods

### Collections, morphology and isolation

Specimens, morphologically similar to *Lophiostoma,* were collected in Chiang Mai and Chiang Rai, Thailand, and brought back to the laboratory in paper bags. Gross morphology was observed using a Motic SMZ 168 dissecting microscope. Vertical sections of ascomata were made by freehand and mounted in water on a slide to observe their microscopic features. Indian ink was used to observe the presence or lack of a mucilaginous sheath surrounding the ascospores. Congo red and cotton blue were used as stains to observe the internal structures and septations. Prepared slides were observed under a Nikon ECLIPSE 80i compound microscope, photographed using a Canon EOS 450D digital camera fitted to the microscope, and preserved in lacto glycerol after photographing. The photographed images were processed using Adobe Photoshop CS5 Extended version 12.0 software (Adobe Systems Inc., The United States). Measurements were determined using Tarosoft Image Frame Work program v. 0.9.7. Single spore isolation was carried to obtain pure culture, using the methods mentioned in Chomnunti et al.^28^ and Senanayake et al*.*^29^ in a Petri-dish containing 2% water agar (WA) and incubated overnight at 20–25 °C. Germinating ascospores were then transferred aseptically onto potato dextrose agar (PDA; 39.0 g/L sterile distilled water, Difco potato dextrose). The production of asexual morphs was facilitated as described in Phookamsak et al.^30^ with the use of sterile bamboo pieces. Culture characteristics were observed over a period of 4 weeks and recorded. Type specimens of the new species are deposited in Mae Fah Luang University Herbarium (MFLU), Chiang Rai, Thailand. The living cultures are deposited in Mae Fah Luang University Culture Collection (MFLUCC). Faces of fungi numbers and Index Fungorum numbers are provided as explained in Jayasiri et al.^27^ and Index Fungorum^31^.

### DNA extraction, amplification, sequencing

Fresh mycelium grown on PDA was scraped and used to extract DNA, following the manufacturer’s standard protocol as described in the Biospin Fungus Genomic DNA extraction kit (BioFlux, Hangzhou, P.R. China). Polymerase chain reactions (PCR) were carried out using the following primers: LR0R and LR5 to amplify the 28s subunit rDNA (LSU)^14,32^, NS1 and NS4 to amplify the 18s subunit rDNA (SSU), ITS4 and ITS5 to amplify the internal transcribed spacers (ITS), EF1-983F and EF1-2218R to amplify the translation elongation factor 1 (TEF), and fRPB2-5F and fRPB2-7cR for RNA polymerase II subunit 2 (RPB2)^33,34^. The amplification reaction was performed in a 25 μl reaction volume containing 2 µl DNA, 12.5 μl 2 × Easy Taq PCR SuperMix polymerase and PCR buffer mix, 8.5 μl Distilled-Deionized-Water (ddH_2_O) and 1 µl of each primer. The thermal cycling program for ITS, LSU, SSU and TEF were as follows: an initialization step of 94 °C for 3 min, followed by 40 amplification cycles of 94 °C for 30 s, annealing step at 55 °C for 50 s, elongation step at 72 °C for 1 min and a final extension step of 72 °C for 10 min. The thermal cycling program for RPB2 amplification was performed following the protocols described in Phookamsak et al.^35^. The quality of the PCR products were checked on 1% agarose gel, stained with ethidium bromide. Sequencing was carried out at Shanghai Sangon Biological Engineering Technology & Services Co., Ltd (Shanghai, P. R. China). Sequence data generated from this study are deposited in GenBank.

### Phylogenetic analyses

Separate ITS, LSU, RPB2, SSU, and TEF DNA sequences were subjected to BLAST search engine tool of NCBI for selection of taxa for subsequent phylogenetic analyses. Newly generated sequences in this study, strains with closest identity in GenBank and related strains in recent publications were selected and included in the sequence dataset (Table S1).

Phylogenetic tree (Fig. 1), the single gene sequence data were compiled using BioEdit v. 7.0.9.0^36^, including the new collections. *Capnodium coffeae* (CBS 147.52) was used as outgroup taxon. Each single gene data set was aligned using MAFFT (http://mafft.cbrc.jp/alignment/server/large.html)^36^, and checked manually using BioEdit^37^. As the topology was similar in each evaluated gene region, a combined alignment of the five loci was compiled into one dataset. Maximum likelihood (ML) and Bayesian Inference (BI) analyses, a partitioned analysis was performed with the following five partitions: ITS, LSU, SSU, RPB2 and TEF sequence data. Maximum-likelihood (ML) analysis was carried out in the CIPRES Science Gateway web server (RAxML-HPC2 on XSEDE^38^), and 1000 rapid bootstrap replicates were run with GTRGAMMA model of nucleotide evolution. Maximum likelihood bootstrap values (MLBS) equal or greater than 60% are presented at each node in the resulting phylogenetic trees. The model of evolution was performed by using MrModeltest 2.3^39^. GTR + I + G model was selected as a best-fit model for the all gene regions used in our analyses. Bayesian inference (BI) analysis was performed in the CIPRES Science Gateway web server (MrBayes on XSEDE v. 3.2.7a^38^). The Markov Chain Monte Carlo sampling (MCMC) analyses, with four chains starting from random tree topology, were run between 10,000,000 generations for each combined dataset. Trees were sampled every 100 generations using a relative burn-in discarding the first 25% of sampled trees^40^**.** Bayesian posterior probabilities equal or greater than 0.90 are given at each node (Fig. 1).

In Fig. 2, maximum parsimony analysis (MP) was performed for the species of *Sublophiostoma* using PAUP (Phylogenetic Analysis Using Parsimony) v. 4.0b10^41^ using a combined dataset of the LSU, SSU, ITS, TEF and RPB2. The heuristic search option with 1,000 random sequences addition and tree-bisection reconnection (TBR) of branch-swapping algorithm were performed. Maxtrees were setup to 1000, branches of zero length were collapsed. Gaps were treated as missing data and all characters were unordered and of equal weight. All multiple and equally parsimonious trees were saved. Descriptive tree statistics for parsimony consistency index (CI), retention index (RI), rescaled consistency index (RC) and homoplasy index (HI) were calculated. The robustness of the most parsimonious tree was evaluated by 1000 bootstrap replications resulting from maximum parsimony analysis, each with ten replicates of random stepwise addition of taxa^42^. The resulting phylogram (the first of 1,000 trees) is presented in Fig. 2. In addition, Bayesian inference (BI) analysis was performed for Fig. 2 following the method mentioned above but with 1,000,000 generations.

Phylograms were visualized with FigTree v1.4.0 program^43^ and reorganized in Microsoft power point (2007) and Adobe Photoshop CS5 Extended version 12.0 software. The finalized alignments and trees were deposited in TreeBASE (http://www.treebase.org), Fig. 1: submission ID: 27,125 (Reviewer access URL: http://purl.org/phylo/treebase/phylows/study/TB2:S27125?x-access-code=836bec690482861d31d89d20881bbd39&format=html).

## Supplementary Information

Supplementary Information
