# Supplementary material for: Introducing a new pleosporalean family Sublophiostomataceae fam. nov. to accommodate Sublophiostoma gen. nov
Source: Sci Rep. 2021 May 4;11:9496. doi: 10.1038/s41598-021-88772-w (PMC8096836; doi:10.1038/s41598-021-88772-w)
Supplement: Supplementary file 1 — Supplementary Information [file 41598_2021_88772_MOESM1_ESM.docx]

**Introducing a new pleosporalean family Sublophiostomataceae fam. nov. to accommodate Sublophiostoma gen. nov.**

Sinang Hongsanan^1,3^, Rungtiwa Phookamsak^2,6,9,10^, Ishani D. Goonasekara^2,3,4^, Kasun M. Thambugala^7,8^, Kevin D. Hyde^2,3^, D. Jayarama Bhat^5^, Nakarin Suwannarach^11,12^ & Ratchadawan Cheewangkoon^1*^

^1^Department of Entomology and Plant Pathology, Faculty of Agriculture, Chiang Mai University, Chiang Mai 50002, Thailand

^2^CAS Key Laboratory for Plant Biodiversity and Biogeography of East Asia (KLPB), Kunming Institute of Botany, Chinese Academy of Science, Kunming 650201, Yunnan, China

^3^Center of Excellence in Fungal Research, Mae Fah Luang University, Chiang Rai 57100, Thailand

^4^School of Science, Mae Fah Luang University, Chiang Rai 57100, Thailand

^5^No. 128/1-J, Azad Housing Society, Curca, P.O. Goa Velha 403108, India

^6^East and Central Asia Regional Office, World Agroforestry Centre (ICRAF), Kunming 650201, Yunnan, China.

^7^Genetics and Molecular Biology Unit, Faculty of Applied Sciences, University of Sri Jayewardenepura, Gangodawila, Nugegoda, Sri Lanka.

^8^Department of Plant and Molecular Biology, Faculty of Science, University of Kelaniya, Kelaniya, Sri Lanka

^9^Honghe Center for Mountain Futures, Kunming Institute of Botany, Chinese Academy of Sciences, Honghe County 654400, Yunnan, China

^10^Centre for Mountain Futures (CMF), Kunming Institute of Botany, Kunming 650201, Yunnan, China.

^11^Research Center of Microbial Diversity and Sustainable Utilization, Chiang Mai University, Chiang Mai 50200, Thailand

^12^Department of Biology, Faculty of Science, Chiang Mai University, Chiang Mai, 50200, Thailand

*email: ratchadawan.c@cmu.ac.th

**Supplementary Table S1.** GenBank accession numbers of the taxa used in the phylogenetic analyses.

| **Species names** | **Strain/Isolate number** | **LSU** | **SSU** | **ITS** | **TEF** | **RPB2** |
| --- | --- | --- | --- | --- | --- | --- |
| *Acrocalymma aquatica* | MFLUCC 11-0208 | JX276952 | JX276953 | JX276951 | - | - |
| *Acrocalymma medicaginis* | CPC 24340 | KP170713 | - | KP170620 | - | - |
| *Acrocalymma pterocarpi* | MFLUCC 17-0926 | MK347949 | MK347840 | MK347732 | MK360040 | - |
| *Aigialus grandis* | BCC 20000 | GU479775 | GU479739 | - | GU479839 | - |
| *Alfoldia vorosii* | CBS 145501 | MK589354 | MK589346 | JN859336 | MK599320 | - |
| *Alternaria alternata* | AFTOL-ID 1610 | DQ678082 | KC584507 | KF465761 | KC584634 | KC584375 |
| *Amniculicola aquatica* | MFLUCC 16-1123 | MK106096 | MK106108 | - | MK109800 | - |
| *Amorocoelophoma cassiae* | MFLUCC 17-2283 | MK347956 | NG_065775 | MK347739 | MK360041 | MK434894 |
| *Amorosia littoralis* | - | AM292055 | AM292056 | AM292047 | - | - |
| *Angustimassarina lonicerae* | MFLUCC 15-0087 | KY496724 | - | KY496759 | - | - |
| *Anteaglonium parvulum* | SMH5223 | GQ221909 | - | - | GQ221918 | - |
| *Anthosulcatispora subglobosa* | MFLUCC 17-2065 | MT214592 | MT226705 | MT310636 | MT394649 | MT394706 |
| *Aquastroma magniostiolata* | CBS 139680 | AB807510 | AB797220 | LC014540 | AB808486 |  |
| *Aquasubmersa japonica* | HHUF:30469 | NG_057138 | NG_062426 | NR_154739 | LC194384 | LC194421 |
| *Aquasubmersa mircensis* | MFLUCC 11-0401 | NG_042699 | NG_061141 | JX276954 | - | - |
| *Ascocylindrica marina* | MD6011 | KT252905 | KT252907 | - | - | - |
| *Ascocylindrica marina* | MF416 | MK007123 | MK007124 | - | - | - |
| *Astragalicola vasilyevae* | MFLUCC 17-0832 | MG828986 | MG829098 | NR_157504 | MG829193 | MG829248 |
| *Astrosphaeriella fusispora* | MFLUCC 10-0555 | KT955462 | KT955443 | - | KT955425 | KT955413 |
| *Atrocalyx acutisporus* | KT 2436 | LC194341 | LC194299 | LC194475 | LC194386 | LC194423 |
| *Bambusicola bambusae* | MFLUCC 11-0614 | JX442035 | JX442039 | JX442031 | - | KP761718 |
| *Berkleasmium crunisia* | BCC 17023 | DQ280271 | - | DQ280265 | - | - |
| *Berkleasmium typhae* | BCC 12536 | DQ280275 | - | DQ280264 | - | - |
| *Biatriospora marina* | CY_1228 | GQ925848 | GQ925835 | - | GU479848 | GU479823 |
| *Brevicollum hyalosporum* | MFLUCC 17-0071 | MG602200 | MG602202 | MG602204 | MG739516 | - |
| *Camarosporidiella caraganicola* | MFLUCCC 14-0605 | KP711381 | KP711382 | KP711380 | - | - |
| *Camarosporium quaternatum* | CPC 31081 | NG_064442 | KY929123 | NR_159756 | KY929201 | - |
| *Camarosporomyces flavigenus* | CBS 314.80 | GU238076 | NG_061093 | MH861266 | - | - |
| *Capnodium coffeae* | CBS 147.52 | MH868489 | DQ247808 | MH856967 | - | KT216519 |
| *Capulatispora sagittiformis* | HHUF 29754 | NG_042319 | NG_060997 | NR_119393 | LC001756 | - |
| *Caryospora minima* | - | EU196550 | EU196551 | - | - | - |
| *Cayospora quercus* | MFLU 18-2151 | NG_066440 | MK347869 | - | - | - |
| *Clypeoloculus akitaensis* | KT 788 | AB807543 | AB797253 | AB809631 | AB808519 | - |
| *Coelodictyosporium rosarum* | MFLUCC 17-0776 | NG_059056 | NG_063674 | MG828875 | MG829195 | - |
| *Coniothyrium palmarum* | CBS 400.71 | JX681084 | EU754054 | MH860184 | - | KT389592 |
| *Corynespora cassiicola* | CBS 100822 | GU301808 | GU296144 | - | GU349052 | GU371742 |
| *Corynespora smithii* | CABI 5649b | GU323201 | - | - | GU349018 | GU371783 |
| *Corynespora torulosa* | CPC 15989 | KF777207 | - | NR_145181 | - | - |
| *Crassiparies quadrisporus* | HHUF 30409 | NG_059028 | NG_061267 | NR_148185 | - | - |
| *Crassiperidium octosporum* | MAFF 246406 | LC373116 | LC373092 | LC373104 | LC373128 | LC373140 |
| *Crassiperidium octosporum* | MAFF 242971 | NG_066389 | NG_065689 | NR_161016 | LC373120 | LC373132 |
| *Cryptoclypeus ryukyuensis* | KT 3534 | LC194347 | LC194305 | LC194481 | LC194392 | LC194429 |
| *Cryptocoryneum japonicum* | HHUF:30482 | NG_059035 | NG_065118 | NR_153938 | LC096144 | LC194438 |
| *Cryptocoryneum pseudorilstonei* | CBS 113641 | NG_059036 | LC194322 | NR_153941 | LC096152 | LC194446 |
| *Cucurbitaria berberidis* | MFLUCC 11-0387 | KC506796 | KC506800 | - | - | - |
| *Cyclothyriella rubronotata* | CBS 141486 | KX650544 | NG_061252 | NR_147651 | KX650519 | KX650574 |
| *Cyclothyriella rubronotata* | CPC 27604 | MH107933 | - | MH107886 | - | - |
| *Cylindroaseptospora leucaenicola* | MFLUCC 17-2424 | MK347966 | MK347856 | NR_163333 | MK360047 | - |
| *Dacampia engeliana* | Hafellner 72868 | KT383791 | - | - | - | - |
| *Dacampia hookeri* | Hafellner 73897 | KT383792 | - | - | - | - |
| *Dacampia hookeri* | Hafellner 75980 | KT383794 | - | - | - | - |
| *Darksidea beta* | CBS 135637 | KP184023 | KP184074 | NR137957 | KP184189 |  |
| *Decaisnella formosa* | BCC 25616 | GQ925846 | GQ925833 | - | GU479851 | GU479825 |
| *Delitschia chaetomioides* | SMH 3253.2 | GU390656 | - | - | GU327753 | - |
| *Delitschia winteri* | AFTOL-ID 1599 | DQ678077 | DQ678026 | - | DQ677922 | DQ677975 |
| *Dendryphion fluminicola* | MFLUCC 17-1689 | MG208141 | - | NR_157490 | MG207992 | - |
| *Dictyocheirospora bannica* | KH 332 | AB807513 | AB797223 | LC014543 | AB808489 | - |
| *Dictyosporium elegans* | NBRC 32502 | DQ018100 | DQ018079 | DQ018087 | - | - |
| *Didymella exigua* | CBS 183.55 | MH868977 | GU296147 | MH857436 | - | - |
| *Didymella rumicicola* | CBS 683.79 | MH873007 | - | KT389503 | - | KT389622 |
| *Didymosphaeria rubi-ulmifolii* | MFLUCC 14-0023 | KJ436586 | KJ436588 | MK646049 | - | - |
| *Dimorphosporicola tragani* | CBS 570.85 | KU728536 | - | KU728497 | KU728577 | - |
| *Dothidotthia_aceris* | MFLUCC 16-1183 | MK751816 | MK751761 | MK751726 | - | - |
| *Falciformispora senegalensis* | CBS 196.79 | NG_057981 | NG_062928 | MH861195 | KF015687 | KF015717 |
| *Falciformispora tompkinsii* | CBS 200.79 | MH872968 | NG_062929 | MH861199 | KF015685 | KF015719 |
| *Fissuroma calami* | MFLUCC 13-0836 | MF588993 | NG_062430 | - | MF588975 | - |
| *Flammeascoma bambusae* | MFLU 11-0143 | NG_059553 | KP753952 | NR_132915 | - | - |
| *Flammeascoma lignicola* | MFLUCC 10-0128b | KT324583 | - | - | - | - |
| *Flavomyces fulophazii* | CBS 135761 | NG_058131 | NG_061191 | NR_137960 | - | - |
| *Foliophoma fallens* | CBS 161.78 | GU238074 | GU238215 | KY940772 | - | KC584502 |
| *Foliophoma fallens* | CBS 284.70 | GU238078 | GU238218 | MH859609 | - | - |
| *Fuscostagonospora cytisi* | MFLUCC 16-0622 | KY770978 | KY770977 | - | KY770979 | - |
| *Fuscostagonospora sasae* | HHUF:29106 | AB807548 | AB797258 | AB809636 | AB808524 | - |
| *Fusculina eucalypti* | CBS 120083 | DQ923531 | - | DQ923531 | - | - |
| *Fusculina eucalyptorum* | CBS:145083 | MK047499 | - | NR_161140 | - | - |
| *Galeaticarpa aomoriensis* | KT 2563 | LC194366 | LC194324 | LC194482 | LC194393 | - |
| *Glonium circumserpens* | CBS 123343 | FJ161200 | - | - | - | - |
| *Glonium stellatum* | ANM32 | GQ221887 | - | - | GQ221926 | - |
| *Gordonomyces mucovaginatus* | CBS 127273 | NG_057941 | - | MH864326 | - | - |
| *Guttulispora crataegi* | MFLUCC 13-0442 | NG_059563 | NG_061203 | KP899134 | KR075161 | - |
| *Halobyssothecium obiones* | MFLUCC 15-0381 | MH376744 | MH376745 | MH377060 | MH376746 | - |
| *Halojulella avicenniae* | BCC 20173 | GU371822 | GU371830 | - | GU371815 | GU371786 |
| *Halojulella avicenniae* | JK 5326A | GU479790 | GU479756 | - | - | - |
| *Halomassarina thalassiae* | JK 5262D | GU301816 | - | - | GU349011 | - |
| *Halotthia posidoniae* | BBH 22481 | GU479786 | GU479752 | - | - | - |
| *Hazslinszkyomyces aloes* | CBS 136437 | KF777198 | - | KF777142 | - | - |
| *Helicascus_kanaloanus* | ATCC_18591 | KX639748 | KX639744 | KX957961 | KX639756 | KX639752 |
| *Helminthosporium velutinum* | L131 | KY984352 | KY984432 | KY984352 | KY984463 | KY984413 |
| *Hermatomyces iriomotensis* | HHUF 30518 | LC194367 | LC194325 | LC194483 | LC194394 | LC194449 |
| *Hermatomyces tectonae* | MFLUCC 14-1140 | KU764695 | KU712465 | KU144917 | KU872757 | KU712486 |
| *Hongkongmyces pedis* | HKU35 | NG_056287 | KF314117 | NR_149338 | - | KF314124 |
| *Hypsostroma caimitalense* | GKM1165 | GU385180 | - | - | - | - |
| *Hypsostroma saxicola* | SMH5005 | GU385181 | - | - | - | - |
| *Hysterium angustatum* | CBS:123334 | FJ161207 | - | - | - | - |
| *Hytereobrevium smilacis* | CBS 114601 | FJ161174 | FJ161135 | - | FJ161091 | FJ161114 |
| *Jalapriya pulchra* | MFLUCC 15-0348 | KU179109 | KU179110 | KU179108 | - | - |
| *Latorua caligans* | CBS 576.65 | NG_058180 | - | MH858723 | - | - |
| *Latorua grootfonteinensis* | CBS 369.72 | NG_058181 | - | - | - | - |
| *Lentithecium clioninum* | HHUF:28199 | NG_059391 | NG_064845 | NR_154137 | AB808515 | - |
| *Lentithecium pseudoclioninum* | HHUF 29055 | NG_059392 | NG_064847 | AB809633 | AB808521 | - |
| *Lepidosphaeria nicotiae* | AFTOL-ID 1576 | DQ678067 | - | - | DQ677910 | DQ677963 |
| *Leptosphaeria cichorium* | MFLUCC 14-1063 | KT454712 | KT454728 | KT454720 | - | - |
| *Leucaenicola aseptata* | MFLUCC 17-2423 | MK347963 | MK347853 | MK347746 | MK360059 | MK434891 |
| *Leucaenicola phraeana* | MFLUCC 18-0472 | MK348003 | NG_065784 | MK347785 | MK360060 | MK434867 |
| *Libertasomyces myopori* | CPC 27354 | NG_058241 | - | KX228281 | - | - |
| *Ligninsphaeria jonesii* | GZCC 15-0080 | KU221038 | - | - | - | - |
| *Ligninsphaeria jonesii* | MFLUCC 15-0641 | NG_059642 | - | - | - | - |
| *Lignosphaeria thailandica* | MFLUCC 11-0376 | KP888645 | - | KP899139 | - | - |
| *Lindgomyces cigarospora* | G619 | KX655804 | KX655805 | KX655794 | - | - |
| *Lindgomyces ingoldianus* | ATCC 200398 | AB521736 | NG_016531 | NR_119938 | - | - |
| *Longiostiolum tectonae* | MFLUCC 12-0562 | KU764700 | KU712459 | KU712447 | - | - |
| *Longipedicellata aptrootii* | MFLU10-0297 | KU238894 | KU238895 | KU238893 | KU238892 | KU238891 |
| *Lophiostoma macrostomum* | KT508 | AB619010 | AB618691 | - | LC001751 | - |
| *Lophiotrema eburnoides* | KT 1424-1 | LC001707 | LC001706 | LC001709 | LC194403 | LC194458 |
| *Macrodiplodiopsis desmazieri* | CBS 140062 | NG_058182 | - | NR_132924 | - | - |
| *Massaria inquinans* | WU 30527 | HQ599402 | HQ599444 | HQ599402 | HQ599342 | HQ599460 |
| *Massarina eburnea* | CBS 473.64 | GU301840 | AF164367 | AF383959 | - | - |
| *Mauritiana rhizophorae* | BCC 28866 | GU371824 | GU371832 |  | GU371817 | GU371796 |
| *Melanomma japonicum* | MAFF 239634 | NG_060360 | NG_065122 | NR_154215 | LC203367 | LC203395 |
| *Misturatosphaeria aurantonotata* | GKM 1238 | NG_059927 | - | - | GU327761 | - |
| *Morosphaeria muthupetensis* | NFCCI4219 | MF614796 | MF614797 | MF614795 | MF614798 | - |
| *Morosphaeria velatispora* | KH221 | AB807556 | AB797266 | LC014572 | AB808532 | - |
| *Multilocularia bambusae* | MFLUCC 11-0180 | KU693438 | KU693442 | KU693446 | - | - |
| *Murilentithecium rosae* | MFLUCC 15-0044 | MG829030 | NG_062439 | MG828920 | - | - |
| *Murispora galii* | MFLUCC 13-0819 | KT709175 | KT709182 | KT736081 | KT709189 | - |
| *Mytilinidion acicola* | EB 0349 | GU323209 | GU323185 | - | - | GU371757 |
| *Mytilinidion californicum* | EB 0385 | GU323208 | GU323186 | - | - | - |
| *Neoascochyta paspali* | CBS 560.81 | MH873129 | NG_062791 | MH861378 | - | KP330426 |
| *Neobambusicola strelitziae* | CBS 138869 | KP004495 | - | KP004467 | MG976037 | - |
| *Neocamarosporium goegapense* | CPC 23676 | KJ869220 | - | KJ869163 | - | - |
| *Neoconiothyrium persooniae* | CBS:143175 | MG386094 | - | MG386041 | - | - |
| *Neohelicascus aquaticus* | MFLUCC 10-0918 | AB807532 | AB797242 | AB809627 | AB808507 | - |
| *Neomassaria fabacearum* | MFLUCC 16-1875 | KX524145 | NG_061245 | - | KX524149 | - |
| *Neomassaria formosana* | NTUCC 17-007 | MH714756 | MH714759 | - | MH714762 | MH714765 |
| *Neomassarina thailandica* | MFLU 11-0144 | NG_059718 | - | NR_154244 | - | - |
| *Neomassarina thailandica* | MFLUCC 17-1432 | MT214467 | MT214420 | MT214373 | - | - |
| *Neomedicopsis prunicola* | CBS:145031 | NG_066330 | - | NR_163368 | - | - |
| *Neopaucispora rosaecae* | MFLUCC 17-0807 | MG829033 | NG_061293 | MG828924 | MG829217 | - |
| *Neophaeosphaeria agaves* | CPC 21264 | KF777227 | - | KF777174 | - | - |
| *Neophaeosphaeria filamentosa* | CBS 102202 | GQ387577 | GQ387516 | JF740259 | GU349084 | GU371773 |
| *Neophaeosphaeria phragmiticola* | KUMCC 16-0216 | MG837009 | NG_065735 | - | MG838020 | - |
| *Neoplatysporoides aloeicola* | CPC 24435 | KR476754 | - | KR476719 | - | - |
| *Neoplatysporoides aloes* | CPC 36068 | MN567619 | - | NR_166316 | - | - |
| *Neopyrenochaeta cercidis* | MFLUCC 18-2089 | MK347932 | MK347823 | MK347718 | - | MK434908 |
| *Neopyrenochaeta telephoni* | FMR 15754 | MH877672 | KR260987 | KM516291 | - | LT717685 |
| *Neopyrenochaetopsis hominis* | UTHSC:DI16-238 | LN907381 | - | LT592923 | - | LT593061 |
| *Neoroussoella bambusae* | MFLUCC 11-0124 | KJ474839 | - | KJ474827 | KJ474848 | KJ474856 |
| *Neotestudina rosatii* | CBS 690.82 | DQ384107 | DQ384069 | - | - | - |
| *Neoyrenochaeta acicola* | CBS 812.95 | GQ387602 | GQ387541 | NR_160055 | - | LT623271 |
| *Nigrograna fuscidula* | CBS 141556 | KX650550 | - | NR_147653 | KX650525 | - |
| *Nigrograna mackinnonii* | CBS 674.75 | GQ387613 | NG_061081 | NR_132037 | KF407986 | KF015703 |
| *Nigrograna obliqua* | CBS 141475 | KX650558 | KX650512 | KX650558 | KX650530 | KX650579 |
| *Noosia banksiae* | CBS 129526 | MH878062 | - | - | - | - |
| *Occultibambusa bambusae* | MFLUCC 13-0855 | KU863112 | - | KU940123 | KU940193 | KU940170 |
| *Occultibambusa jonesii* | GZCC 16-0117 | KY628322 | KY628324 | - | KY814756 | KY814758 |
| *Parabambusicola bambusina* | KH 139 | AB807537 | AB797247 | LC014579 | AB808512 | - |
| *Paradictyoarthrinium aquatica* | MFLUCC 16-1116 | NG_064501 |  | NR_158861 | - | - |
| *Paradictyoarthrinium diffractum* | MFLUCC 13-0466 | KP744498 | KP753960 | KP744455 | - | KX437764 |
| *Paralophiostoma hysterioides* | PUFNI 17617 | MT912850 | - | MN582758 | - | - |
| *Paramassaria samaneae* | MFLU 17-1551 | MK108190 | MK108187 | - | MK105747 | - |
| *Parapyrenochaeta acaciae* | CBS 141291 | KX228316 | - | KX228265 | - | LT717686 |
| *Parapyrenochaeta protearum* | CBS 131315 | JQ044453 | - | JQ044434 | - | LT717683 |
| *Pararoussoella mukdahanensis* | MFLUCC 11-0201 | NG_059671 | - | NR_155722 | - | - |
| *Paratrimmatostroma kunmingensis* | KUN-HKAS 102224A | MK098196 | MK098204 | MK098192 | MK098208 | - |
| *Paucispora quadrispora* | KT 843 | AB619011 | AB618692 | LC001734 | LC001755 | - |
| *Periconia delonicis* | MFLUCC 17-2584 | NG_068611 | NG_065770 | - | - | MK434901 |
| *Periconia pseudodigitata* | KT 1395 | AB807564 | AB797274 | LC014591 | - | - |
| *Phaeomycocentrospora cantuariensis* | CBS 112.24 | MH866267 | - | MH854763 | - | - |
| *Phaeoseptum mali* | MFLUCC 17-2108 | MK625197 | - | MK659580 | MK647990 | MK647991 |
| *Phaeoseptum terricola* | MFLUCC 10-0102 | MH105779 | MH105780 | MH105778 | MH105781 | MH105782 |
| *Phaeosphaeria oryzae* | CBS 110110 | KF251689 | GQ387530 | KF251186 | - | KF252193 |
| *Phaeosphaeriopsis glaucopunctata* | MFLUCC 13-0265 | KJ522477 | KJ522481 | KJ522473 | MG520918 | - |
| *Phaeosphaeriopsis triseptata* | MFLUCC 13-0271 | KJ522479 | KJ522484 | KJ522475 | MG520919 | KJ522485 |
| *Phoma herbarum* | CBS 615.75 | EU754186 | NG_061066 | KF251212 | KF253168 | KP330420 |
| *Phoma neerlandica* | CBS 134.96 | KT389753 | - | KT389535 | - | KT389661 |
| *Platystomum compressum* | MFLUCC 13-0343 | KP888643 | KP899129 | - | KR075165 | - |
| *Pleiochaeta carotae* | CPC 27452 | KY905663 | - | KY905669 | - | - |
| *Plenodomus salviae* | MFLUCC 13-0219 | KT454717 | KT454732 | KT454725 | - | - |
| *Pleohelicoon fagi* | MFLUCC 17-2538 | MK348036 | MK347925 | MK347816 | - | MK434851 |
| *Pleohelicoon richonis* | CBS 282.54 | - | AY856952 | MH857332 | - | - |
| *Pleomonodictys descalsii* | FMR 12716 | KY853522 | - | KY853461 | - | - |
| *Polyschema terricola* | CBS 301.65 | NG_057767 | NG_061058 | NR_160100 | - | EF204487 |
| *Preussia funiculata* | CBS 659.74 | GU301864 | GU296187 | - | GU349032 | GU371799 |
| *Preussia lignicola* | CBS 363.69 | DQ384098 | - | GQ203783 | - | - |
| *Prosthemium betulinum* | CBS 127468 | MH875932 | - | MH864496 | - | - |
| *Pseudoasteromassaria spadicea* | MFLUCC 15-0973 | KY522724 | KY522725 | KY522726 | - | - |
| *Pseudoastrosphaeriella bambusae* | MFLUCC 11-0205 | KT955475 | KT955455 | - | KT955437 | KT955414 |
| *Pseudoastrosphaeriella longicolla* | MFLUCC 11-0171 | KT955476 | - | - | KT955438 | KT955420 |
| *Pseudoastrosphaeriella thailandensis* | MFLUCC 11-0144 | KT955478 | KT955457 | NR_154244 | KT955440 | KT955416 |
| *Pseudoberkleasmium chiangmaiense* | MFLUCC 17-1809 | MK131260 | - | MK131259 | MK131261 | - |
| *Pseudoberkleasmium pandanicola* | KUMCC 17-0178 | MH260304 | MH260344 | MH275071 | - | - |
| *Pseudochaetosphaeronema pandanicola* | MFLUCC 16-0272 | MH260316 | MH260356 | MH275082 | - | - |
| *Pseudocoleodictyospora tectonae* | MFLUCC 12-0385 | KU764709 | NG_061232 | NR_154338 | - | KU712491 |
| *Pseudocoleodictyospora thailandica* | MFLUCC 12-0565 | KU764701 | NG_062417 | NR_154337 | - | KU712494 |
| *Pseudodidymosphaeria spartii* | MFLUCC 13-0273 | KP325436 | KP325438 | KP325434 | - | - |
| *Pseudohelminthosporium clematidis* | MFLUCC 17-2086 | MT214567 | MT226683 | MT310612 | MT394627 | MT394690 |
| *Pseudolophiostoma vitigenum* | HHUF 26930 | AB619015 | NG_060998 | NR_154374 | LC001761 | - |
| *Pseudomassariosphaeria grandispora* | CBS 613.86 | GU301842 | GU296172 | - | - | EF165042 |
| *Pseudoneoconiothyrium rosae* | MFLUCC 15-0052 | MG829032 | MG829138 | MG828922 | - | - |
| *Pseudopyrenochaeta lycopersici* | FMR 15746 | EU754205 | NG_062728 | NR_103581 | - | LT717680 |
| *Pseudopyrenochaeta terretris* | FMR 15327 | LT623216 | - | LT623228 | - | LT623287 |
| *Pseudotetraploa longissima* | HC 4933 | AB524612 | AB524471 | AB524796 | AB524827 | - |
| *Pseudotrichia mutabilis* | SMH 1541 | GU385209 | - | - | - | - |
| *Pseudoxylomyces elegans* | KT 2887 | AB807598 | AB797308 | LC014593 | AB808576 | - |
| *Pyrenochaetopsis leptospora* | CBS 101635 | GQ387627 | NG_063097 | JF740262 | MF795881 | LT623282 |
| *Pyrenochaetopsis tabarestanensis* | IBRC M 30051 | KF803343 | NG_065034 | NR_155636 | - | - |
| *Pyrenophora phaeocomes* | AFTOL-ID 283 | NG_027575 | JN940960 | JN943649 | DQ497607 | DQ497614 |
| *Quadricrura bicornis* | yone 153 | AB524613 | AB524472 | AB524797 | AB524828 | - |
| *Quercicola fusiformis* | MFLUCC 18-0479 | MK348009 | MK347898 | MK347790 | MK360085 | MK434864 |
| *Quercicola guttulospora* | MFLUCC 18-0481 | MK348010 | MK347899 | MK347791 | MK360086 | - |
| *Quixadomyces cearensis* | HUEFS 238438 | MG970695 | - | NR_160606 | - | - |
| *Raghukumaria keshaphalae* | PUFD65 | MK282438 | MK282440 | MK282439 | - | - |
| *Ramusculicola thailandica* | MFLUCC 13-0284 | KP888647 | KP899131 | KP899141 | KR075167 | - |
| *Roussoella nitidula* | MFLUCC 11-0634 | KJ474842 | - | KJ474834 | KJ474851 | KJ474858 |
| *Salsuginea phoenicis* | MFLU 19-0015 | MK405280 | - | - | MK404650 | - |
| *Salsuginea ramicola* | KT 2597.2 | GU479801 | GU479768 | - | GU479862 | GU479834 |
| *Salsuginea ramicola* | KT 2597.1 | GU479800 | GU479767 | - | GU479861 | GU479833 |
| *Seltsamia ulmi* | CBS 143002 | MF795794 | MF795794 | MF795794 | MF795882 | MF795836 |
| *Shiraia bambusicola* | GZAAS2.0629 | KC460980 | - | GQ845415 | - | - |
| *Shiraia bambusicola* | GZAAS2.0703 | KC460981 | - | GQ845412 | - | - |
| *Spirosphaera cupreorufescens* | A20 | AY616236 | - | AY616232 | - | - |
| *Splanchnonema platani* | CBS 222.37 | KR909316 | KR909318 | MH855895 | KR909319 | KR909322 |
| *Sporormia fimetaria* | UPS:Dissing Gr.81.194 | GQ203729 | - | GQ203769 | - | - |
| *Stagonospora paludosa* | CBS 135088 | KF251760 | - | KF251257 | - | KF252262 |
| *Stemphylium herbarum* | CBS 191.86 | GU238160 | GU238232 | NR111243 | KC584731 | DQ247794 |
| *Striatiguttula nypae* | MFLUCC 18-0265 | MK035992 | MK035977 | MK035969 | MK034432 | MK034440 |
| *Striatiguttula phoenicis* | MFLUCC 18-0266 | MK035995 | MK035980 | MK035972 | MK034435 | MK034442 |
| ***Sublophiostoma thailandica*** | **MFLUCC 11-0165** | **KX534213** | **KX534219** | **MW136258** | **KX550078** | **MW088715** |
| ***Sublophiostoma thailandica*** | **MFLUCC 11-0172** | **KX534214** | **KX534220** | **MW136255** | **KX550079** | **MW088716** |
| ***Sublophiostoma thailandica*** | **MFLUCC 11-0174** | **KX534215** | **KX534221** | **MW136265** | **-** | **MW088717** |
| ***Sublophiostoma thailandica*** | **MFLUCC 11-0185** | **KX534216** | **KX534222** | **MW136275** | **KX550080** | **MW088718** |
| ***Sublophiostoma thailandica*** | **MFLUCC 11-0207** | **KX534212** | **KX534218** | **MW136257** | **KX550077** | **MW088714** |
| ***Sublophiostoma thailandica*** | **MFLUCC 12-0006** | **KX534217** | **KX534223** | **-** | **KX550081** | **MW088719** |
| *Submerspora variabilis* | MFLUCC 17-2360 | MN913682 | - | MT627683 | - | - |
| *Subplenodomus violicola* | CBS 306.68 | MH870849 | GU238231 | MH859138 | - | - |
| *Sulcatispora acerina* | KT 2982 | LC014610 | LC014605 | LC014597 | LC014615 | - |
| *Sulcatispora berchemiae* | KT 1607 | AB807534 | AB797244 | AB809635 | AB808509 | - |
| *Sulcosporium thailandica* | MFLUCC 12-0004 | KT426563 | KT426564 | MG520958 | - | - |
| *Teichospora trabicola* | C134 | KU601591 | - | KU601591 | KU601601 | KU601600 |
| *Tetraplosphaeria sasicola* | KT 563 | AB524631 | AB524490 | AB524807 | AB524838 | - |
| *Thyridaria acaciae* | CBS 138873 | NG_058127 | - | KP004469 | - | - |
| *Thyridaria broussonetiae* | TB1 | KX650568 | KX650515 | KX650568 | KX650539 | KX650586 |
| *Thyrostroma compactum* | CBS 335.37 | KY905664 | - | KY905670 | KY905681 | - |
| *Torula aquatica* | MFLUCC 16-1115 | MG208146 | - | MG208167 | - | MG207977 |
| *Torula pluriseptata* | MFLUCC 14-0437 | KY197855 | KY197862 | MN061338 | KY197875 | KY197869 |
| *Tremateia arundicola* | MFLU 16-1275 | KX274248 | KX274254 | KX274241 | KX284706 |  |
| *Trematosphaeria grisea* | CBS 332.50 | NG_057979 | NG_062930 | NR_132039 | KF015698 | KF015720 |
| *Trematosphaeria pertusa* | CBS 122368 | NG_057809 | FJ201991 | NR_132040 | KF015701 | FJ795476 |
| *Tzeanania taiwanensis* | NTUCC 17-006 | MH461121 | MH461127 | MH461124 | MH461131 | - |
| *Tzeanania taiwanensis* | NTUCC 17-005 | MH461120 | MH461126 | MH461123 | MH461130 | - |
| *Uzbekistanica rosae-hissaricae* | MFLUCC 17-0819 | MG829087 | NG_061297 | MG828975 | MG829242 | MG829262 |
| *Vaginatispora aquatica* | MFLUCC 11-0083 | KJ591576 | KJ591575 | KJ591577 | - | - |
| *Verruculina enalia* | BCC 18402 | GU479803 | GU479771 | - | GU479864 | GU479836 |
| *Westerdykella ornata* | CBS 379.55 | GU301880 | GU296208 | NR_103587 | GU349021 | GU371803 |
| *Wicklowia aquatica* | CBS 125634 | MH875044 | NG_061099 | - | - | - |
| *Wicklowia* sp*.* | MFLUCC:18-0373 | MK637644 | MK637643 | - | - | - |
| *Xenodidymella applanata* | CBS 195.36 | KT389764 | - | MH855770 | - | - |
| *Xenopyrenochaetopsis pratorum* | CBS 445.81 | GU238136 | NG_062792 | MH861363 | - | KT389671 |
